# Chloroquine could be used for the treatment of filoviral infections and other viral infections that emerge or emerged from viruses requiring an acidic pH for infectivity

**DOI:** 10.1002/cbf.3182

**Published:** 2016-03-21

**Authors:** Hephzibah Akpovwa

**Affiliations:** ^1^ Cardiff School of Biosciences Cardiff University Cardiff UK

**Keywords:** Ebola virus, Marburg virus, chloroquine, hydroxychloroquine, necrosis and apoptosis

## Abstract

Viruses from the Filoviridae family, as many other virus families, require an acidic pH for successful infection and are therefore susceptible to the actions of 4‐aminoquinolines, such as chloroquine.

Although the mechanisms of action of chloroquine clearly indicate that it might inhibit filoviral infections, several clinical trials that attempted to use chloroquine in the treatment of other acute viral infections – including dengue and influenza A and B – caused by low pH‐dependent viruses, have reported that chloroquine had no clinical efficacy, and these results demoted chloroquine from the potential treatments for other virus families requiring low pH for infectivity.

The present review is aimed at investigating whether chloroquine could combat the present Ebola virus epidemic, and also at exploring the main reasons for the reported lack of efficacy. Literature was sourced from PubMed, Scopus, Google Scholar, reference list of articles and textbooks – Fields Virology (Volumes 1and 2), the cytokine handbook, Pharmacology in Medicine: Principles and Practice, and hydroxychloroquine and chloroquine retinopathy.

The present analysis concludes that (1) chloroquine might find a place in the treatment of Ebola, either as a monotherapy or in combination therapies; (2) the ineffectiveness of chloroquine, or its analogue, hydroxychloroquine, at treating infections from low pH‐dependent viruses is a result of the failure to attain and sustain a steady state concentration sufficient to increase and keep the pH of the acidic organelles to approximately neutral levels; (3) to successfully treat filoviral infections – or other viral infections that emerge or emerged from low pH‐dependent viruses – a steady state chloroquine plasma concentration of at least 1 µg/mL(~3.125 μM/L) or a whole blood concentration of 16 μM/L must be achieved and be sustained until the patients' viraemia becomes undetectable. These concentrations, however, do not rule out the efficacy of other, higher, steady state concentrations – although such concentrations might be accompanied by severe adverse effects or toxicities. The feasibility of the conclusion in the preceding texts has recently been supported by a subsequent study that shows that amodiaquine, a derivative of CQ, is able to protect humans infected with Ebola from death.

## Introduction

The Filoviridae family – containing Ebola and Marburg viruses – comprises enveloped viruses with nonsegmented, negative‐sense RNA genomes,^1^, ^2^, ^3^ each of which consists of seven genes forming the structural and nonstructural proteins.^1^, ^2^ Of all filoviruses, the currently circulating Zaire species of Ebola virus (EboV) is the most virulent, with a mortality rate ranging from 59 to 88%.^1^, ^2^

The peplomers of the EboV species are composed of trimerized heterodimers of glycoproteins 1 and 2, which are heavily glycosylated with both N‐linked and O‐linked glycans, and contain abundant α(2‐6) and/or α(2‐3) linked sialic acids.^1^, ^2^, ^3^ These peplomers have broad tropism for a variety of host cells and organs as a result of their ability to bind either specifically or nonspecifically to various cell surface molecules.^1^, ^2^, ^4^, ^5^, ^6^, ^7^, ^8^ However, despite this broad tropism, infection by filoviruses greatly depends on the acidic pH of the organelles internalizing the virus,^1^, ^2^, ^8^, ^9^, ^10^ and therefore this characteristic can be exploited therapeutically.

Filovirus binding to surface molecules on the plasma membrane of susceptible cells – such as tissue macrophages, monocytes, dendritic cells, endothelial cells and hepatocytes – leads to the internalization of virions into vesicles which traffic through the endosomal pathway.^1^, ^2^, ^8^, ^9^, ^10^ To successfully infect susceptible cells, the virus requires endosomal acidification and the cleavage of the glycoprotein 1 segment of the peplomer by host endosomal proteases (active in acidic pH)^1^, ^2^, ^8^, ^9^, ^10^ and, without this acidification and cleavage, the infection is abrogated.^1^, ^2^, ^10^ Therefore, therapeutic agents targeting endosomal acidification (and hence pH‐dependent proteases), could be beneficial in combating the present African EboV epidemic.

Successful infection induces the local and systemic release of varying amounts of cytokines, chemokines, reactive oxygen species, nitric oxide and other mediators,^1^, ^2^, ^11^, ^12^, ^13^, ^14^ and eventually results in generalized cell death.^1^, ^2^, ^11^, ^12^, ^13^ If a patient's immune system is able to control the infection, the patient recovers^1^, ^2^, ^11^, ^12^, ^13^, ^14^ – although convalescence is prolonged, and recovering patients have been shown to produce infectious virus many months after symptoms have disappeared.^1^, ^2^ However, if a patient's immune system is unable to control the infection, further cycles of infection in susceptible cells and organs occur, leading to further release of the mediators mentioned in the preceding texts, with consequently massive cell death.^1^, ^2^, ^11^, ^12^, ^13^ This is manifested in fatal cases as extensive cell death in many organs – including the liver, spleen, lymph nodes, kidney and adrenal glands – and coagulopathies, which are revealed as disseminated intravascular coagulation, haemorrhages, petechiae, ecchymosis, congestion and uncontrolled bleeding at venipuncture sites.^1^, ^2^, ^11^, ^12^, ^13^ The mode of infection of filoviruses and the associated pathologies may uncover an Achilles' heel to a therapeutic weaponry based on 4‐aminoquinolines such as chloroquine (CQ).

### Therapeutically exploiting the tropism of chloroquine for the acidic organelles in the treatment of filoviral infections

Chloroquine is a weak base, commonly used in the treatment of malaria and autoimmune diseases such as rheumatoid arthritis and systemic lupus erythematosus.^15^, ^16^, ^17^, ^18^, ^19^, ^20^, ^21^, ^22^, ^23^ It is readily absorbed when administered by either oral or parenteral routes,^15^, ^16^, ^17^, ^18^, ^19^, ^20^, ^21^, ^22^, ^23^ becoming highly concentrated in tissues such as the adrenal glands, liver, spleen and kidney^15^, ^16^, ^17^, ^18^, ^23^ – tissues suffering extensive necrosis in fatal filoviral infections.^1^, ^2^, ^4^ In the cells of these and other tissues, CQ becomes concentrated in acidic organelles such as the endosomes, lysosomes and Golgi vesicles,^16^, ^17^, ^18^, ^24^, ^25^, ^26^, ^27^, ^28^, ^29^, ^30^ thereby increasing their pH^24^, ^25^, ^26^, ^27^, ^28^, ^29^, ^31^ and leading to the dysfunction of several enzymes, e.g. those required for the proteolytic processing and the post‐translational modification of proteins.^10^, ^24^, ^25^, ^27^, ^31^, ^32^, ^33^, ^34^ CQ also inhibits the production of several immunological mediators, the excessive release of which contributes to autoimmune diseases such as systemic lupus erythematosus and rheumatoid arthritis.^18^, ^24^ Therefore, these CQ properties should be considered in the treatment for filoviral infections and their associated pathologies.

The CQ‐increased pH of the acidic organelles has been shown to inhibit several viruses – including influenza A and B, SARS coronavirus, hepatitis A virus and the Borna disease virus – which all require a low pH for entry.^24^, ^34^, ^35^, ^36^, ^37^, ^38^, ^39^, ^40^ This suggests that CQ could also inhibit filoviruses' entry into the cytoplasm of susceptible cells and thereby abrogate their infection, since this is dependent on endosomal acidification and the activities of several host endosomal proteases. CQ might also inhibit the assembly and budding of filoviruses – which partly require the late endosome in their assembly.^1^, ^2^

Accordingly, the dysfunction of enzymes, e.g. some glycosyltransferases, caused by CQ or hydroxychloroquine (HCQ), as a direct result of the increased pH and/or structural changes in the Golgi apparatus, has been shown to inhibit not only the glycosylation of SARS coronavirus,^34^ but also that of HIV‐1 gp120, thereby leading to the production of noninfectious virions or virus with decreased infectivity.^31^, ^33^, ^40^, ^41^ This mechanism has been invoked to explain the decrease in viral load that was observed when patients with HIV‐1 were orally administered HCQ at 800 mg/day for 8 weeks.^31^, ^42^ These results, though obtained with nonrelated viruses, could suggest that CQ, if given at the correct dosage, might inhibit the glycosylation of EboV peplomers, which is more pronounced than that occurring with HIV‐1.^1^, ^2^, ^3^, ^43^ Since the GP of filoviruses is the only protein involved in initiating infection,^1^, ^2^, ^5^, ^6^, ^8^ and cytotoxicity is dependent on its expression,^1^, ^2^, ^5^ inhibiting its glycosylation could potentially (1) inhibit EboV tropism for a broad variety of host cells and organs; (2) lead to the production of noninfectious or decreased infectivity virus (as seen with HIV‐1)^31^, ^33^; and (3) decrease Ebov pathogenicity. Impaired glycosylation could therefore save time for the adaptive immune response, which normally fails in fatal cases,^1^, ^2^, ^11^ to be established and deal with the infection.

### Therapeutically exploiting the immunomodulatory properties of chloroquine in the treatment of filoviral infections

Apart from therapeutically exploiting the increased pH and dysfunction of enzymes caused by CQ, the immunomodulatory properties of this drug could also be exploited therapeutically in the treatment of some of filoviral infection‐associated pathologies. Several studies have suggested that the multiple organ failure and hypovolemic shock seen in fatal cases are likely a result of both direct infection and destruction of susceptible cells, such as endothelial cells, and the effect of pro‐inflammatory cytokines, chemokines and other mediators released from infected and activated cells, such as monocytes and macrophages.^1^, ^2^, ^4^, ^6^, ^10^, ^11^, ^12^, ^44^

One cytokine strongly implicated in these pathologies is TNF‐α,^1^, ^2^, ^7^, ^12^, ^13^ which is able to increase the permeability of endothelial cells, as shown in experiments conducted with the human umbilical vein.^1^, ^2^, ^7^ It is also able – in humans injected with a recombinant form – to induce a sustained activation of blood coagulation and also cause tissue injury and shock.^45^, ^46^, ^47^ The role of TNF‐α in the pathologies and fatalities associated with filoviral infections is further confirmed by the fact that (1) intramuscular administration of anti‐TNF‐α serum, after 4–7 days postinfection, is able to reduce the circulating concentration of TNF‐α and protect 50% of infected rodents from death^1^, ^2^; (2) patients who recovered from infection with Zaire EboV (ZEboV) in two recent outbreaks in Gabon had a transient increase in the plasma concentration of TNF‐α at the onset of infection – which then decreased during the course of the infection. Conversely, fatal cases display an increased and sustained concentration of TNF‐α.^12^ In these fatalities, highly increased concentrations of soluble tumour necrosis factor receptors were also detected.^12^ Taken together, these observations clearly show that a therapeutic agent like CQ, which is able to prevent the activation of macrophages and also inhibit the secretion of TNF‐α from various cells at clinically relevant concentrations,^24^, ^48^, ^49^, ^50^, ^51^, ^52^ could be of some benefit in the treatment of filoviral infections.

Another cytokine likely to be implicated in Ebola pathogenesis – especially the massive apoptosis or lymphopenia associated with fatal cases of EboV infections – is IFN‐γ.^11^, ^12^, ^13^ It has been reported that IFN‐γ is able to increase cellular sensitivity to apoptosis by upregulating the expression of Fas and Fas ligand,^53^ and in all fatal cases of EboV infection, the expression of IFN‐γ, Fas and Fas ligand was shown to be upregulated.^11^ Another mechanism by which IFN‐γ might – at least in part – account for the massive apoptosis detected in Filovirus infections is induction of production by monocytes/macrophages of neopterin and its derivative, 7‐8‐dihydroneopterin.^12^, ^54^ It has been reported that neopterin and 7‐8‐dihydroneopterin induce apoptosis in a rat alveolar cell line,^54^ and that the plasma concentration of neopterin significantly and progressively increases throughout the disease course of all fatal cases of ZEboV infections – but not in survivors.^12^ Therefore, the production of neopterin and 7‐8‐dihydroneopterin upon IFN‐γ stimulation might also contribute to the massive apoptosis seen in fatal cases of EboV infections.^11^, ^12^, ^13^

Thus, since the massive apoptosis seen in all fatal cases of EboV‐infected patients is associated with the production of IFN‐γ, neopterin and 7‐8‐dihydroneopterin, and TNF‐α,^11^, ^12^, ^13^ a therapeutic agent like CQ, having been shown, at clinically relevant concentrations, to inhibit the production of IFN‐γ, TNF‐α and neopterin from various cells,^24^, ^48^, ^49^, ^50^, ^51^, ^52^ could be of great benefit in the treatment of patients infected with the present ZEboV or other filoviruses.

### Sufficient steady state chloroquine concentrations may be needed to achieve its therapeutic effects

Although the above mechanisms of the action of CQ and the various *in vitro* studies suggest that CQ may exert some benefit in infections from viruses that require an acidic pH for infectivity and/or are heavily glycosylated and evoke a detrimental immune activation, several clinical trials that attempted to use CQ or HCQ in the prevention or treatment of several viral infections – including influenza A and B, HIV‐1 and dengue viral infections – have reported that CQ or HCQ either had undetectable/moderate clinical efficacy or were actually detrimental (Table 1).^42^, ^55^, ^56^, ^57^, ^58^, ^59^, ^60^, ^61^, ^62^, ^63^, ^64^, ^65^, ^66^

**Table 1:** Outcome of several clinical trials, anecdotal trials and an animal study on the efficacy of CQ or HCQ in the treatment of low pH‐dependent or pH‐independent viral infections

Type of 4‐AQ, route	Dose and duration	Infection/Type of study	Plasma drug concentration (ng/mL)	Outcome of trial	*n*	Reference
HCQ, po	800 mg/d; 8 weeks	HIV‐1/CT	Steady state of 27 to 1000.4; mean of 316.3	Moderate efficacy; patient with highest concentration showed the best response.	40	42
CQ, po	250–500 mg/d; 8 weeks	HIV‐1/CT	Not stated	Significant reduction of immune activation, but no effect on viral load	12	58
CQ, po	200 mg/d; 16 weeks	HIV‐1/CT	Not stated	No efficacy	20	59
HCQ, po	400 mg/d; 48 weeks	HIV/CT	Not stated	Increased viral load in nine of the patients	42	60
HCQ, po	400 mg/d; 6 months	HIV‐1/CT	Not stated	Significant reduction of immune activation	20	61
CQ, po	500 mg/d for 1 week, then once a week until the 12th week	Influenza‐A/CT	Not stated	No efficacy	724	55
CQ, po	Day 1 = 600 mg Day 2 = 300 mg Day 3 = 300 mg	Dengue/CT	Not stated	No efficacy	153	56
CQ, po	500 mg/d; 3 days	Dengue/CT	Not stated	Improved dengue‐related symptoms, which returned after medication was stopped	19	57
CQ, parenteral	Not stated	Ebola/Anecdotal	Not measured	Patient fever resolved rapidly and remained afebrile for 6 days, but died latter from symptoms indicative of Ebola infection	1	62
CQ	Not stated	Ebola/Anecdotal	Not measured	No efficacy stated	1	63
CQ, ip	90 mg/kg; twice daily for14 days	Ebola/Animal study	Steady state whole blood concentration of 2.5 µg/mL	85% survival rate	20	64

po, oral administration; ip, intraperitoneal; CT, clinical trial; d, day; AQ, aminoquinoline; CQ, chloroquine; HCQ, hydroxychloroquine.

It is possible to hypothesize that these mixed or disappointing results may be attributable to not knowing – and thus not achieving – the therapeutic steady state plasma or whole blood CQ concentration necessary for exerting its therapeutic effects (refer to the succeeding texts). In order for CQ to be used for the treatment of infections from viruses that require an acidic pH for infectivity and/or are heavily glycosylated and depend on their envelop glycoprotein for infectivity, the therapeutic steady state plasma or whole blood concentration must be achieved and sustained until the patient's viraemia becomes undetectable. In the succeeding texts, I summarize some of the evidence that can be used to determine the necessary therapeutic, steady state plasma or whole blood CQ concentration.

Firstly, several *in vitro* studies have shown that the optimal uptake of CQ in several cell types isolated from different animals is in the range of 10–20 μM, with concentrations >~30 μM, causing less uptake.^26^, ^30^

Secondly, the whole blood CQ EC_50_ of 17.7 μM/L is considered to be an *in vivo* threshold of CQ toxicity since it is a level associated with significant cardiovascular effects in patients with CQ poisoning,^67^ whilst patients having concentrations of 16 μM/L were shown to have no significant cardiovascular events.^67^

Thirdly, out of all the clinical trials summarized in Table 1, only the trial that administered the highest HCQ dose, for a duration sufficient to attain a steady state (0.08 to 2.98 μM/L, mean = 0.94 μM/L), reported a moderate improvement in several of the patients.^42^ Of all the patients, only the one with the highest HCQ level (2.98 μM/L) (which is approximately equivalent to the *in vitro* CQ EC_50_ of ~3 μM shown to inhibit HIV‐1 replication^33^) had the best response to HCQ. This patient's absolute CD4+ T‐cells increased from 200 to 400 cells/mm^3^, the plasma levels of HIV‐1 RNA decreased from 225 to 135 cpm, the percentage of CD4+ T‐cells increased from 11% to 34%, and there was also a significant improvement in mitogen responses.^42^ Although HIV does not depend on the acidic organelles for infectivity and is less glycosylated in comparison to EboV,^1^, ^2^, ^3^, ^43^ HCQ, at this steady state concentration, is nevertheless able to inhibit HIV replication by affecting gp120 glycosylation.^31^, ^33^, ^42^

Fourthly, the steady state CQ/HCQ concentrations of 1 µg/mL (~3.125 μM/L) and ~16 μM/L detected in plasma and whole blood respectively, have been shown both *in vivo* and *in vitro* to inhibit viral replication and the overproduction of some immunological mediators associated with the pathologies of many viral infections and some autoimmune diseases.^22^, ^48^, ^64^, ^68^, ^69^, ^70^

I conclude that (1) a 16 μM/L transient or steady state whole blood concentration of CQ most likely has no significant cardiovascular events^22^, ^42^, ^67^, ^68^, ^69^; (2) ~16 μM/L steady state whole blood concentration of CQ/HCQ is able to inhibit viral replication, glycosylation and the over production of some immunological mediators associated with some viral infections^22^, ^33^, ^34^, ^35^, ^38^, ^39^, ^42^, ^64^, ^67^, ^68^, ^69^, ^70^; (3) the optimal uptake of CQ in humans is likely to lie within the range of 10–20 μM/L. Therefore, such a steady state CQ concentration could be safe and sufficient to raise and maintain the acidic organelles' pH to a level approximately neutral, thereby inhibiting viral replication by mechanisms such as inhibition of endosomal proteases, inhibition of the fusion of viral membrane with host cells plasma membranes and inhibition of viral glycosylation.

In order to achieve the recommended therapeutic steady state plasma or whole blood CQ concentrations, the use of slow, continuous and constant IV infusion could be recommendable, since (1) it could be more efficient in achieving the stated therapeutic concentration and (2) it is more controlled, given the low cardiovascular safety margin of CQ.^18^, ^21^

Having recommended the use of constant IV infusion, suggesting a precise dose and an infusion rate for filoviral infection treatment (as in the treatment of malaria), though seemingly reasonably, is, however, impossible at this stage. This is primarily because of the interindividual differences in the pharmacokinetics of CQ absorption.^69^, ^71^

## Conclusion

I conclude that 1 µg/mL (~3.125 μM/L) or 16 μM/L steady state CQ concentration in plasma or whole blood respectively, could be used for the treatment of filoviral and other infections by viruses requiring an acidic pH for infectivity, and that these concentrations need to be sustained until the patients' viraemia becomes undetectable. These stated concentrations, however, do not rule out the efficacy of other, higher, steady state concentrations – though such concentrations might be accompanied by severe adverse effects or toxicities. Other subsequent research has supported the conclusion in the preceding texts by confirming that a derivative of CQ has some protective effect against Ebola infection in humans.^72^

## Conflicts of Interest

The author declares no conflict of interest.
